# Social Network Extraction and Analysis Based on Multimodal Dyadic Interaction

**DOI:** 10.3390/s120201702

**Published:** 2012-02-07

**Authors:** Sergio Escalera, Xavier Baró, Jordi Vitrià, Petia Radeva, Bogdan Raducanu

**Affiliations:** 1 Centre de Visió per Computador, Campus UAB, Edifici O, Bellaterra, 08193 Barcelona, Spain; E-Mails: xbaro@uoc.edu (X.B.); jordi@cvc.uab.es (J.V.); petia@cvc.uab.es (P.R.); bogdan@cvc.uab.es (B.R.); 2 Department Matemàtica Aplicada i Anàlisi, Universitat de Barcelona, Gran Via 585, 08007 Barcelona, Spain; 3 Estudis d’Informàtica, Multimèdia i Telecomunicació, Universitat Oberta de Catalunya, Rambla del Poblenou 156, 08018 Barcelona, Spain

**Keywords:** social interaction, audio/visual data fusion, influence model, social network analysis

## Abstract

Social interactions are a very important component in people’s lives. Social network analysis has become a common technique used to model and quantify the properties of social interactions. In this paper, we propose an integrated framework to explore the characteristics of a social network extracted from multimodal dyadic interactions. For our study, we used a set of videos belonging to New York Times’ Blogging Heads opinion blog. The Social Network is represented as an oriented graph, whose directed links are determined by the Influence Model. The links’ weights are a measure of the “influence” a person has over the other. The states of the Influence Model encode automatically extracted audio/visual features from our videos using state-of-the art algorithms. Our results are reported in terms of accuracy of audio/visual data fusion for speaker segmentation and centrality measures used to characterize the extracted social network.

## 1. Introduction

Social interactions play a very important role in people’s daily lives. Whether face-to-face or electronic (via e-mails, SMS, online communities, *etc*.), they represent the main communication channel people use to strengthen their inter-personal ties. Social interactions can be defined as a dynamic sequence of social actions between individuals who modify and adapt their behavior according to those of their partners. Social action is a concept that refers to the interaction between individuals in society and it is used to observe how certain behaviors are modified in certain conditions [1]. Although from a psychologic point of view the study of social interactions has captured the interest of researchers long time ago [2], it is only nowadays that an increasing interest has been manifested in automatic analysis of social interactions.

Most of the existing communication systems used by people nowadays, including some popular online communities (Facebook, YouTube, Flickr, Digg, Twitter), infer the social interactions based on explicit input analysis. However, recently, a new paradigm has been introduced by Pentland [3], according to which, face to face social interactions can be inferred based on an implicit input analysis. This computational paradigm is based on the analysis of social signals in the form of nonverbal (nonlinguistic) communication: facial expressions, voice tonality, hand and body gestures, *etc*. The roots of a computational model for social signal processing can be found in social psychology [4]. User studies demonstrated that nonlinguistic social signals are particularly powerful for analyzing and predicting human behaviour. There is enough implicit information contained in nonlinguistic communication (voice prosody, face and body gestures) such that we could get a hint regarding the outcome of an interaction process (e.g., agreement/disagreement in a conversation, *etc*.).

Social Network Analysis (SNA) [5,6] has been developed as a tool to model the social interactions in terms of a graph-based structure. “Nodes” represent the “actors” who make the subject of the analysis (persons, communities, institutions, corporate data) and the “links” represent the specific type of interdependencies (friendship, familiarity, knowledge exchange, financial transactions) that exist between “nodes”. SNA uncovers the implicit relationships between “actors” and provides understanding of the underlying social processes and behaviors. It has become a widely used technique in a variety of application areas such as the WWW, organizational studies [7,8], security domain [9,10], *etc*. We could identify two main areas of interest where SNA has been applied: sub-group identification based on blogs or data-sharing websites and functional role recognition in work-groups.

The application of SNA techniques to the first category is relatively new. In [11], authors used SNA for the purpose of analyzing the structure of online hate group blogs. In [12], authors examine how communities can be discovered through interconnected blogs as a form of social hypertext. Another study of the online social networks is reported in [13], where the authors consider the evolution of structure within two large online communities: Yahoo!360° and Flickr. The Youtube community has been also analyzed in [14], where the authors use the links among videos of different users to define a social network useful for ranking videos and improve the quality of content retrieval applications.

Regarding the application of SNA to the second category, some work is reported in [15]. The model proposed allowed not only the study of direct effects of functional role, status, and communication role on individual performance, but also indirect effects through individual centrality. A similar work, which addresses the problem of assessing both the individual and group performance, is presented in [16]. In [17,18], authors offer a different application of SNA to workgroups: speaker role recognition in meetings. With their approach, they are able to identify up to six different roles. The study of SNA-based role identification has also been analyzed in [19].

**Figure 1.**
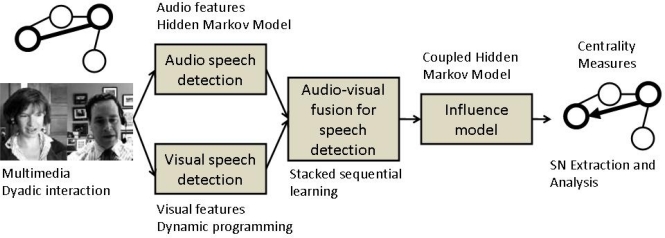
Block diagram of our integrated framework for Social Network extraction and analysis.

Our work is inline with Pentland’s computational paradigm, pretending to serve as a “proof-of-concept”. The paper presents an integrated framework for extraction and analysis of a social network from multimodal dyadic interactions. First, speaker segmentation is performed through an audio/visual fusion scheme using state-of-the-art algorithms. Second, in order to model the relationship in the dyadic interactions, we employed the Influence Model [20] whose states encode the integrated audio/visual data. Third, the social network is represented as a directed graph, whose links are estimated based on the “influence” one person has over the other. The dyadic interactions which make the object of our study belong to the publicly available New York Times’ Blogging Heads opinion blog [21]. The whole process is depicted in Figure 1.

The paper is structured as follows. Section 2 presents our approach for audio/video feature extraction and fusion, based on the particularities of our problem. Section 3 presents the extraction of the Social Network, including a brief recall of the Influence Model. In Section 4, we explain the analysis performed on the extracted network. Finally, Section 5 contains conclusions and guidelines for future work.

## 2. Audio-Visual Cues Extraction and Fusion

Audio cues are the primary source of information that can provide useful evidence for speech production. However, they cannot easily distinguish a user who is speaking from the others in a noisy environment, where several speakers talk simultaneously. Alternatively, visual cues can be useful in deciding whether somebody is the active talking person by analyzing his/her lips movement. However, visual cues alone cannot easily distinguish a speaker from an active listener, who may be just smiling or nodding without saying anything. For this reason, combining both audio and visual cues, we expect to obtain an increased robustness in the speaker diarization process.

### 2.1. Audio Cue

In order to obtain the audio structure, we use a diarization scheme based on the approach presented in [22]. These features correspond to the state-of-the-art on audio descriptions, which have been successfully applied in several audio analysis applications [23–25]. The process is described next:

**Description:** The input audio is analyzed using a sliding-window of 25 ms, with an overlap of 10 ms between two consecutive windows, and each window is processed using a short-time Discrete Fourier Transform, mapping all frequencies to the mel scale. Finally, the Discrete Cosine Transform (DCT) is used in order to obtain the first 12 MFCC coefficients. Those coefficients are complemented with the energy coefficient and the dynamic features *delta* and *delta-delta*, which correspond to the first and second time-derivatives of cepstral coefficients.
**Speaker segmentation:** Once the audio data is properly codified by means of those features, next step is to identify the segments of the audio source which corresponds to each speaker. A first coarse segmentation is generated according to a Generalized Likelihood Ratio, computed over two consecutive windows of 2.5 s. Each block is represented using a Gaussian distribution with full covariance matrix over the extracted features. This process produces an over segmentation of the audio into homogeneous small blocks. Then, a hierarchical clustering is applied over the segments. We use an agglomerative strategy, where initially each segment is considered as a cluster, and at each iteration the two most similar clusters are merged, until the Bayesian Information Criterion (BIC) stopping criterion is met. As in the previous step, each cluster is modeled by means of a Gaussian distribution with full covariance matrix, and centroid distance is used as link similarity. Finally, a Viterbi decoding is performed in order to adjust the segment boundaries. Cluster are modelled by mean of a one state HMM having as observation model GMM with diagonal covariance matrices.

### 2.2. Visual Speech Classification

Visual cue extraction refers to the segmentation of mouth region and classification of mouth-area appearance in speaking and non-speaking patterns.

#### 2.2.1. Feature Extraction

**Figure 2.**
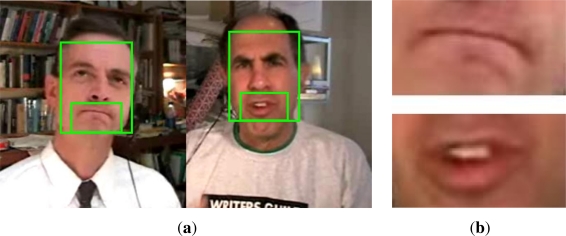
(**a**) Face and mouth detection and (**b**) segmented mouth regions.

In our case, the data we are working with consists of dyadic conversations in which the speakers are in near-frontal view with respect to the camera. For this kind of scenarios, we use a face detector in order to limit the search area for the mouth region. One of the most robust and fast face detectors is the one proposed by Viola and Jones [26]. An example of face detection from a snapshot of the video depicting dyadic interactions is shown in Figure 2(a). From the detected regions, the mouth regions are extracted as the down-middle area of the detected face (Figure 2(b)).

The face detector provides a robust detection of faces in a near frontal-view of the head to respect the camera. Given the nature of the considered data set, this technique provides robust results. However, in some situations where the speaker moves the head, the face is not properly detected since occlusions are present. In those cases, we ignore the search of the mouth regions. However, this effect only affects less than 0.5% of the video frames. In contrast, the method is invariant to scale, and robust against the motion produced by abrupt movements of the head given its real-time computation and the high frame rate. Moreover, we included temporal constraints to avoid false positive mouth detections in case that more than one face is detected because of the presence of background artifacts. In those non-frequent cases, only the closest detected mouth region compared to previous detections is considered, and no other compensations of head motion are required.

Next step consists in the extraction of discriminative features from the mouth region. In this case, the Histogram of Oriented Gradients (HOG) is one of the preferred descriptors because of its tolerance to illumination changes and noise [27]. In our case, the HOG descriptor codifies the mouth region as an histogram of orientations based on the grey-level gradient vectors of pixels within the detected mouth region. These orientations are grouped into bins, removing low gradient magnitudes. This representation offers a high discrimination power to split lips orientations from speech and non-speech patterns in the classification stage.

#### 2.2.2. Speech Classification

Once we have segmented and described the mouth regions over time, we define the visual speaker diarization as a one-class classification problem. Note that we want to discriminate between speaking and non-speaking patterns. Since the former ones have more intra-class variabilities, we are more interested in modelling the non-speaking patterns, and then, classify those temporal patterns that are far away in terms of distance as the speaking patterns. For this purpose, we take advantage of dynamic programming to match temporal series. In particular, we apply Dynamic Time Warping (DTW).

The goal of DTW [28,29] is to find an alignment warping path between two time series *Q* = {*q*_1_, . . ., *q_n_*} and *C* = {*c*_1_, . . ., *c_m_*}. In order to align these two sequences, a *n* × *m* matrix is designed, where the position (*i, j*) of the matrix contains the distance between *q_i_* and *c_j_*. The Euclidean distance is the most frequently applied. Then, a warping path *W* = {*w*_1_, . ., *w_T_*}, max(*m, n*) ≤ *T* < *m* + *n* + 1 is defined as a set of “contiguous” matrix elements that defines a mapping between *Q* and *C*. We are interested in the final warping path that minimizes the warping cost:

(1) $$\mathrm{DTW}(Q,\;C)=\text{min}\left\{\frac{1}{T}\;\sqrt{\sum_{t=1}^{T}w_{t}}\right\}$$

where *T* compensates for the different lengths of the warping paths. This path can be found very efficiently using dynamic programming to evaluate the following recurrence which defines the cumulative distance *γ*(*i, j*) as the distance *d*(*i, j*) found in the current cell and the minimum of the cumulative distance of the adjacent elements:

(2) γ(i,j)=d(i,j)+min{γ(i−1, j−1), γ(i−1,j), γ(i,j−1)}

**Figure 3.**
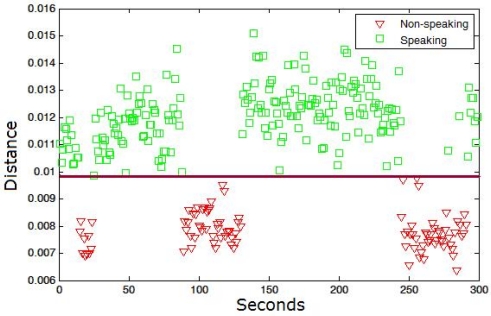
Result of a one-class classification process for an excerpt of five minutes conversation. The legend shows the true label of the samples. Samples are linearly separable using the DTW-based one-classifier.

In our case, the set of model sequences {*C*} is computed using the HOG descriptors of non-speaking mouth regions in *m* consecutive frames. The set {*C*} is obtained by supervised labelling using a reduced set of training samples. Afterwards, the classification of samples as speaking/non-speaking patterns is performed by the extraction of the set of queries {*Q*} (each query fixed to length *m* in our case) from the test samples with some overlapping percentage among consecutive queries, and their alignment to all non-speaking time series {*C*}. If a minimum of *k* samples from {*C*} has a warping cost inferior to a given cost threshold *T* for a given query *Q*, then we classify *Q* as a non-speaking pattern, otherwise, *Q* is classified as speaking pattern. Figure 3 shows an example of the data sequences {*Q*} classification for a speaker within a conversation. The square points are the true labels corresponding to the speaking patterns, and the triangle points to the non-speaking ones. A threshold near 0.01 in the training step correctly splits both patterns in the testing step.

### 2.3. Audio-Video Fusion: Stacked Sequential Learning

Once we have performed the classification of audio and video sequences in speaking and non-speaking patterns, we want to integrate both cues in order to improve the performance of the diarization process. Since our data is characterized by temporal coherence, we use sequential learning, which can deal with the fusion of audio-video features at the same time that includes temporal knowledge in the classification process. This is done by considering the predicted labels of the neighborhood samples as new features for a second learning procedure. In this way, useful temporal relations help the audio-visual fusion to improve final speech classification.

**Figure 4.**
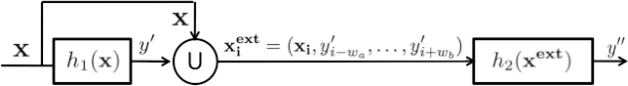
Stacked Sequential Learning scheme.

Sequential learning is a machine learning technique that deals with temporal coherence among data samples. The basic idea of stacked sequential learning is to create an extended data set that joins the original training data features with the predicted labels considering a neighborhood around the example. Figure 4 shows a block diagram of the SSL method. The basic SSL method uses a five-fold cross-validation on the training set to obtain the predicted set *Y*′ and considers a sliding window of length *w* with origin in the prediction of the current example to extend its features. That is, for each example in the training set *x_i_* ∈ **X**, the predicted values *y*′*_i_* ∈ *Y*′ are obtained and joined creating an extended example $x_{i}^{\mathrm{ext}}=\left(x_{i},\;y_{i-w_{a}}^{\prime },\ldots ,y_{i+w_{b}}^{\prime }\right)\in X^{\mathrm{ext}}$, where the number of added features is *w* = *w_a_* + *w_b_* + 1. The extended training set is used to train a second classifier that is expected to capture the sequentiality of the data.

In our case, we resize the vector of visual features to the audio sampling size. Once the vectors fit in size, the combined feature vector is used to train the first classifier *h*_1_. From the output of this classifier over the training data, a neighborhood *w* of predicted labels is included as extra feature for each data point, and a second classifier *h*_2_ is trained. As a result of this procedure, we take into account both audio and visual features together and their temporal relations in the training stage.

## 3. Social Network Extraction and Analysis

The proposed social network is represented as a directed graph. This graph is designed by means of an influence model from the previous audio-visual speech detection methodology and analyzed using different centrality measures.

### 3.1. Network Extraction: The Influence Model

**Figure 5.**
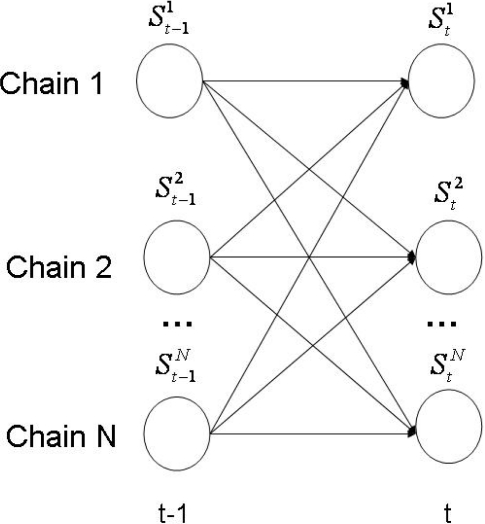
The Influence Model architecture.

The Influence Model (InfModel) [20] is a tool developed to quantitatively analyze a group of interacting agents. In particular, it can be used to model human behavior in a conversational setting. In this context, the participants and their corresponding interactions are modelled through a coupled Hidden Markov Model (HMM). In Figure 5, we offer a visual representation of this architecture.

The model is completely defined by a parametrization scheme that represent the influence of one chain over the others. More concrete, given *N* participants, the multi-process transition probability $P\left(S_{t}^{i}|S_{t-1}^{1},\ldots ,S_{t-1}^{N}\right)$ is approximated only by the transition probability $P\left(S_{t}^{i}|S_{t-1}^{j}\right)$, where *t* represents the time stamp. With this convention, the multi-process transition could be expressed now as:

(3) $$P\left(S_{t}^{i}|S_{t-1}^{1},\ldots ,S_{t-1}^{N}\right)=\sum_{j}\alpha_{\mathrm{ij}}P\left(S_{t}^{i}|S_{t-1}^{j}\right)$$

In other words, the state of chain *i* at time *t* is conditioned only by the state of chain *j* at time *t*−1. The *α_ij_* parameters that appear in the equation above are referred as “influences”, because they are constant factors that tell us how much the state transitions of a given chain depend on a given neighbor.

In the case of conversations, a more intuitive interpretation of the *α* parameters, in a nonverbal setting, would be the following. When we talk to other people we are influenced by their style of interaction. It is a known fact that some persons are more influential than others. In some cases, this causes us to change our natural style and to adopt an attitude closer to our counterpart, tending to become a more active or an equal partner. In some other cases, if we are not affected by our counterpart’s attitude, we will probably tend to maintain our natural expressive style.

**Figure 6.**
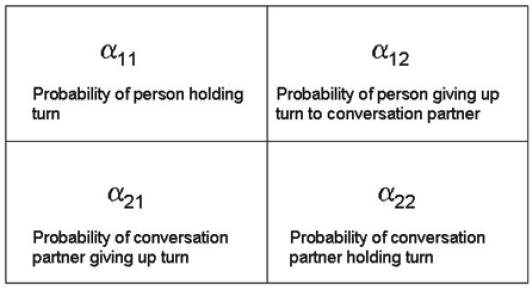
The significance of *α* parameters in the case of a two-person conversation.

When we try to quantify these influences, what we actually do is estimating the transition probabilities for individuals, based on their turn hold and their turn taking with their conversation partner. A graphical representation of the *α* parameters is given in Figure 6.

In its current implementation, the InfModel is able to model interactions between pairs of participants, but it is not able to model the joint effect of several chains together. The learning algorithm for the InfModel is based on constrained gradient descent. For our experiment, we estimated the InfModel based on voicing features (*i.e.*, speaking/non-speaking segments), resulted from the integration of audio/video modalities.

Thus, our social network will be created by estimating the influence one person has over the other from dyadic interactions.

### 3.2. Network Analysis

In social network analysis, a common measure to assess a person’s position in a group is centrality [6]. Several centrality measures exist, which are used to quantify different aspects of a network. Some of them take into account just that if there is a link between two nodes. Others are based on the links’ weight (as a way to quantify the “distance” between two nodes). In our case, the weight values are given by the *α* coefficients from Equation (3). The most common centrality measures are: degree, closeness, betweenness, and eigenvector.

***Degree centrality*** refers to which person is more active by counting the number of connections to other persons. In other words, this means which person is able to communicate directly with the others. In directed graphs, this centrality measure has two components: **in-degree centrality** (number of incoming connections) and **out-degree centrality** (number of outgoing connections). A high in-degree value reflects a person’s availability to receive more information (to be influenced) by others. On the other hand, a high out-degree value reflects a person’s ability to influence the others.

***Closeness centrality*** is based on the geodesic distance between one person and the other in the network. It shows the facility of one person to communicate with the other. Nodes with small centrality values mean that they are “close” to each other. In other words, we expect that the smaller the centrality value is, the higher the influence of the node in the network is.

***Betweenness centrality*** measures how important a person is in bridging two different parts of a network. The removal of such a person (node) could create a breach in the network, which will ultimately lead to a loss of network cohesion. This kind of nodes are very influential in the network topology.

***Eigenvector centrality*** is a measure of the importance of a node in a network. A person’s position in the network is influenced by the other persons position. In other words, a person’s position (influence) in the network increases due to people with high position (influence).

## 4. Experimental Results

In this section we report some experimental results for the integrated framework previously described. We first assess the performance of our proposed scheme for audio/visual feature fusion. Afterwards, we present some centrality measures computed for the social network extracted using the InfModel based on audio/visual features.

Before the presentation of the results, we make a brief description of the data, methods, and validation protocol used in the experiments.

***Data***: The data used in our experiments consists of dyadic interactions from the publicly available New York Times’ Blogging Head opinion blog [21]. The videos show close-ups of two persons talking in front of a webcam about a given topic (most common, politics). One of the persons is the “anchor” and the other one the “guest”. The character of the conversation is totally informal, so the audio signal is somehow monotonic and there are no significant variations in voice energy and speaking rate. In a limited number of videos, we could see that speakers interrupt each other quite often. In the most of the cases, however, the conversations are characterized by long turn-takings and almost the absence of overlapping speech fragments. The average duration of the analyzed conversations is 30 min.

From the whole set of dyadic conversations in the blog, we collected a subset of 17 videos from 15 different people. It is important to remark that this selection has been done taking into account the most active people of the blog. Moreover, the number of conversations selected for each speaker is proportional to his/her activity in the blog. The people featuring in the videos also are somehow connected. This selection criteria is important since it shows the general structure of the most active people in the blog. The remaining participants who do not appear in our selection have a very sporadic participation and form small isolated non-connected sub-graphs in the social network. This selection criteria is important in order to apply the centrality measures described at the previous section, since they will not be applicable over random selection of conversations which do not represent the general structure of the blog activity.

***Validation protocol***: We apply ten-fold cross-validation, saving ten rounds of 90% of the data for training and 10% of the data for testing. The division of the data is done so that at each round the tested users are not considered in the training set. For each video, we show the mean speaker diarization performance by comparing the visual cue alone, audio cue alone, and the result of the audio/visual feature fusion process. The comparison is done taking into account the ground truth segmented from the stereo audio data. Moreover, we look for statistical significance of the obtained performances using Friedman and Nemenyi statistics [30]. Centrality measures are also computed over the designed social network.

### 4.1. Audio-Video Fusion Results

The audio clustering methodology returns a vector of labels where each label corresponds to a possible speaking cluster, including the non-speaking cluster and obtaining different number of clusters for each conversation. Thus, we can not obtain a direct performance in the audio speech segmentation step. However, the fusion with stacked sequential learning associates the audio cluster labels to the corresponding speakers or non-speaking patterns based on the information provided by the video cue features. As we have the stereo and mono audio files of the conversations, we can directly use the stereo files to define the ground truth data and the mono data to perform the experiments. Then, we can measure the robustness of our system when dealing with only one audio channel where different subjects speak, as is the case of real non-controlled applications.

**Figure 7.**
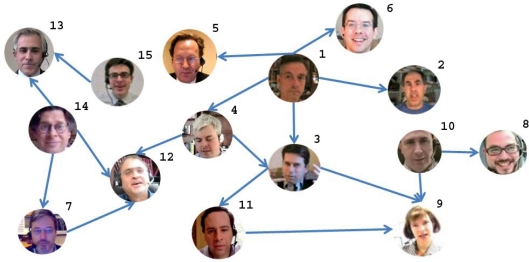
Social Network showing participant labels and influence direction.

Table 1 shows the video, audio, and audio-video fusion mean segmentation results (A-V) comparing with the ground truth data. Each row of the table corresponds to a conversation. The first column identifies the subjects that participate in each conversation (see Figure 7). The labels identifying the subjects and the conversations are graphically shown in the social network of Figure 7. The best performance for both speakers of each conversation is marked in bold. Note that in most cases the fusion methodology considerably improves the video as well as the audio classification, obtaining high accurate predictions. Only in six from the 34 speakers classification, the fusion is not able to improve the video or audio results. Significant performance improvements are obtained with the fusion methodology in more of the 80% of the cases (28 of 34 subjects). An interesting point is that those people that appear in different conversations used to maintain their speech/non-speech discriminability, since they used to act in a similar way.

In order to compare the performances provided for each of the three feature representations, the last row of Table 1 also shows the mean rank of each strategy considering the 17 different data. The rankings are obtained estimating each particular ranking $r_{i}^{j}$ for each data sequence *i* and each feature representation *j*, and computing the mean ranking *R* for each configuration as $R_{j}=\frac{1}{N}\sum_{i}r_{i}^{j}$, where *N* is the total number of data sets.

In order to reject the null hypothesis that the measured ranks differ from the mean rank, and that the ranks are affected by randomness in the results, we use the Friedman test. The Friedman statistic value is computed as follows:

(4) $$X_{F}^{2}=\frac{12N}{k(k+1)}\;\left[\sum_{j}R_{j}^{2}-\frac{k{\left(k+1\right)}^{2}}{4}\right]$$

In our case, with *k* = 3 feature representations to compare, $X_{F}^{2}=23.05$. Since this value is undesirable conservative, Iman and Davenport proposed a corrected statistic:

(5) $$F_{F}=\frac{(N-1)X_{F}^{2}}{N(k-1)-X_{F}^{2}}$$

Applying this correction we obtain *F_F_* = 33.72. With 3 methods and 17 experiments, *F_F_* is distributed according to the *F* distribution with 2 and 32 degrees of freedom. The critical value of *F* (2, 32) for 0.05 is 3.23. As the value of *F_F_* = 33.72 is higher than 3.23 we can reject the null hypothesis.

Once we have checked for the non-randomness of the results, we can perform an a post hoc test to check if one of the configurations can be statistically singled out. For this purpose we use the Nemenyi test. The Nemenyi statistic is obtained as follows:

(6) $$\mathrm{CD}=q_{\alpha }\sqrt{\frac{k(k+1)}{6N}}$$

In our case with *k* = 3 system configurations to compare and *N* = 17 experiments (data sets) the critical value for a 90% of confidence is *CD* = 0.4. As the ranking of the proposed audio-vidual fusion methodology does not intersect with any rank for that value of the *CD*, we can state that our proposal is statistically significant to the rest of system configurations in the presented experiments.

**Figure 8.**
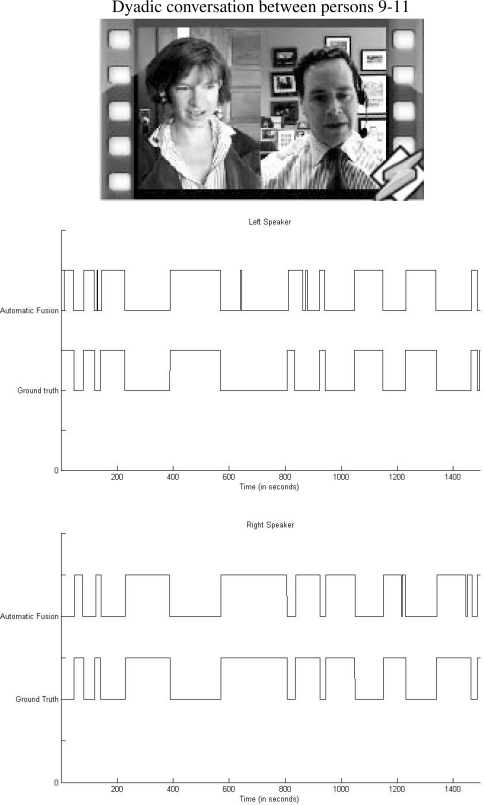
Comparison between speaking prediction with the fusion methodology and ground truth vectors.

We present in a graphic illustration the comparison between ground truth segmentation and automatic fusion scheme corresponding to a fragment of the dyadic conversation depicted in the top of Figure 8. Middle and bottom images of the same figure correspond to the left and right person in the dialog, respectively. Observe the high visual correlation among vectors.

### 4.2. Centrality Measures Results

As a result of the audio/visual fusion scheme previously introduced, we obtained a binary vector whose values represent the speaking/non-speaking states for each person. These vectors are fed into the InfModel in order to get the *α* coefficients (from Equation (3)) which encode the influence values for each person. We used the InfModel implementation which comes with the MIT library for speech processing [31]. Based on the *α* coefficients, we extracted the graph of inter-personal relations: the direction of links reflects the influence of one person over the other. In other words, the links are weighted by the *α* coefficients. An “A→B” link can be interpreted as “A has influence over B”. The resulting graph is depicted in Figure 7.

Note that in this graph only the links with the highest weights (*α* coefficients) are represented. The lack of a link between two persons means that these persons do not interact at all. The number which appears on the upper-right part of each node (face circle) represents person’s number.

Based on the *α* coefficients, we constructed the associated sociomatrix which has been subsequently used in the computation of several centrality measures (An alternative way to characterize the sociomatrix is represented by cliques identification. The term of clique has been imported from the graph theory, and in the context of social networks the interpretation of a clique is “people sharing similar views, values or opinions”. Since our social network is sparse, the maximal clique is of size 2 and it is represented by the following groups of nodes: 14, 7, 12, 4, 1, 3 and 3, 11, 9. The cliques identification is more suitable for highly connected graphs, because they could offer a hint about the degree of coherence that exists in subgroups.): degree (with its two versions, in-degree and out-degree), closeness, betweenness, and eigencentrality. The computed measures are summarized in Table 2. We represented in bold characters the highest values of these measures.

The conclusions we can extract from this table, in some cases, are straightforward. If we are interested, for instance, in the most important node (person) in the network, this is by far the node 1. This is confirmed by out-degree, in-degree, and eigenvector centrality measures. At the same time, we could say that the person designated by node 1 is the most influential person in the group: he influences up to four persons, but nobody else is able to influence him.

If we are interested in the person acting as a cohesion factor for the group (or a hub), this is represented by node 4. This fact is confirmed by the betweenness centrality. We can identify up to 3 subgroups that are bridged by this node: (1,2,5,6), (7,12,13,14,15) and (3,8,9,10,11). For this reason, the role of node 4 in this network is crucial, but his relevance is different from the node 1. For instance, if he is removed from the group, the network structure is severely damaged, practically being split in 3 parts. On the other hand, if we remove node 1, indeed, we face obviously a loss, but the network structure is not affected too much.

From the same table, we can also deduce that node 8 is the most irrelevant component of the group, fact confirmed by all centrality measures we used. If we remove it from the network, the effect is null. On the other hand, nodes 9 and 12 are the weakest, in the sense that they are influenced by all the surrounding neighbors (in-degree centrality measure).

Based on the closeness centrality measures, we can infer that person 9 is the most influenced. On the other hand, person 15 has the most influencing potential.

In all the other cases, some aspects might not be that obvious and a disagreement between measures might happen. This can be explained due to the unsupervised nature of the network extraction process. As we have mentioned before, the dyadic interaction takes place in an informal manner. For this reason, it is impossible to establish, even using human annotators, an absolute ground truth of the interactional process (who has more influence over whom), because in our approach we take into account only the non-verbal analysis, making total abstraction of the conversation content.

## 5. Conclusions

In this paper, we presented an integrated framework for automatic extraction and analysis of a social network from implicit input (multimodal dyadic interactions), based on the integration of audio/visual features using state-of-the-art algorithms. After the extraction of audio and visual cues, we performed a fusion of audio/video data, based on Stacked Sequential Learning, in order to increase the robustness of the speaker segmentation process. Subsequently, the feature vector consisting of the audio/video data has been fed into InfModel in order to determine the influence between persons in the dyadic interaction. Based on the resulting relationships, we built the structure of the social network. Finally, we applied some SNA specific measures (different types of centrality) in order to extract some characteristics of the discovered network.

The main characteristic of the described approach and the proposed application is that it considered for analysis a set of videos depicting people interacting freely, in realistic conditions, not being obliged to follow any pre-arranged script. A positive aspect of the current settings is the use of simple equipment necessary to gather the data (just a webcam and a microphone), keeping this way user intrusiveness at a minimum level. We believe that these factors make our approach very attractive: being simple (not imposing any constraints on people behaviors or the recording conditions) it could be easily reproduced for other real-life scenarios (either indoor or outdoor situations). This is in obvious contrast with some other approaches (such as [32]) which make use of high-tech equipment for the purpose of studying multimodal nonverbal interactions.

A limitation of our approach is the lack of an absolute ground truth. Due to the character of the conversations (informal), it is almost impossible, even for human experts, to assess the real outcome of the interaction process. However, our framework is intended to serve as a “proof-of-concept” of some aspects from social psychology (e.g., human behavior can be quantified and the position of a person in a group could be inferred as a result). Even if our approach has been validated on a reduced dataset, we do not think the size has a significant influence on the conclusions drawn from our experiments.

A potential application of our approach is its use in collaborative working environments. They could offer very useful insights of the actual role each person is playing in the group (based on their activity level or involvement degree) and the group structure per se (is it a coherent group or not). On the other hand, social networks can be used to study how the informational flow propagates in a group or small communities. This will allow distant users to become better integrated into ongoing discussions, and thus improve distance-separated social interaction, teamwork, and social networking. In this context, it is more difficult to quantify these aspects based on explicit input (exchange of messages) due to privacy concerns. But an implicit input analysis would through a more clear perspective of the interactional patterns.

In the future, we plan to extend the current approach and study the problem of social interactions to a larger scale and in different scenarios. Starting from the premise that people’s lives are more structured than it might seem with a naked eye, we plan to study long-term interactions between persons, with the aim to discover underlying behavioral patterns present in our day-to-day existence. For this purpose, we plan to use some wearable, sophisticated sociometric devices, able to record audio, video, and location of the subjects.

## Figures and Tables

**Table 1.** Video, Audio, and A–V speaking classification.

**Conversation**	**Video**		**Audio**		**A–V**	

	**Left**	**Right**	**Left**	**Right**	**Left**	**Right**
1–2	66.58	58.36	75.77	68.32	**81.90**	**78.87**
1–3	58.01	**75.82**	63.90	64.23	**72.90**	72.52
1–4	68.37	78.50	56.38	57.35	**85.48**	**79.16**
1–5	88.99	72.50	88.80	**88.14**	**89.02**	84.02
1–6	69.51	61.86	79.14	80.31	**91.47**	**90.72**
9–3	82.63	61.95	83.83	73.92	**97.88**	**80.75**
9–10	65.01	63.71	96.79	**96.88**	**96.92**	65.44
3–11	65.77	74.91	83.84	86.72	**92.40**	**93.58**
4–3	75.35	64.09	79.10	82.07	**80.05**	**91.73**
4–12	94.13	75.36	**94.70**	91.46	94.21	**93.36**
13–15	70.96	71.95	76.43	77.22	**97.36**	**95.18**
13–14	**61.99**	65.11	43.10	74.97	56.56	**95.24**
12–14	86.20	64.02	88.90	72.28	**90.25**	**88.31**
12–7	97.75	85.26	97.13	92.54	**97.82**	**98.51**
8–10	61.44	55.93	79.18	88.29	**92.56**	**95.09**
9–11	67.09	66.88	89.72	91.37	**97.80**	**94.98**
7–14	55.88	63.54	91.37	**70.17**	**96.09**	60.12

Mean Rank	2.82		2.01		1.17	

**Table 2.** Centrality Measures.

**Node No.**	**Out-degree**	**In-degree**	**Closeness**	**Betweenness**	**Eigencentrality**
1	5	0	0.5344	0.7582	**0.7264**
2	0	1	0.4628	0	0.1278
3	2	2	0.7898	0.7692	0.3241
4	2	1	0.7510	**0.9341**	0.4321
5	0	1	0.5339	0	0.0152
6	0	1	0.3985	0	0.2832
7	1	1	0.5052	0	0.1224
8	0	1	0	0	0
9	0	**3**	**0.8228**	0.5165	0.0437
10	2	0	0.5375	0.1429	0.0187
11	1	1	0.6177	0	0.1084
12	0	**3**	0.6453	0.8352	0.2159
13	0	2	0.4897	0.2747	0.0131
14	3	0	0.5344	0.6264	0.0896
15	1	0	0.3434	0	0.0063
